# Late-onset peritoneal recurrence of advanced gastric cancer 20 years after primary resection

**DOI:** 10.1186/1477-7819-8-104

**Published:** 2010-11-24

**Authors:** Yoshinaga Okugawa, Yuji Toiyama, Yasuhiro Inoue, Susumu Saigusa, Minako Kobayashi, Koji Tanaka, Yasuhiko Mohri, Chikao Miki, Masato Kusunoki

**Affiliations:** 1 Department of Gastrointestinal and Pediatric Surgery, Mie University Graduate School of Medicine, Tsu, Mie, Japan

## Abstract

Late onset of peritoneal recurrence of gastric cancer more than 10 years after surgery is extremely rare, and only three cases have been reported. We present the case of a 61-year-old man who was diagnosed finally with peritoneal recurrence of gastric cancer 20 years after primary curative resection. As a result of small-bowel obstruction caused by peritoneal recurrence, diverting ileostomy with partial ileal resection was performed. The resected specimen revealed tubular adenocarcinoma that resembled the primary gastric cancer. The clinical course after the second operation was unfavorable and systemic chemotherapy had no effect. He died at 62 years of age, 21 years and 7 months after initial gastrectomy. Immunohistochemical analysis using antibodies against proliferating cell nuclear antigen (PCNA), Ki-67, and p53 was performed to investigate the phenotype of primary and recurrence cancer. Protein expression of proliferation markers such as PCNA and Ki-67 was down-regulated, but p53 was overexpressed at the site of recurrence. These data suggest that late peritoneal recurrence has a low proliferation rate and is resistant to chemoradiotherapy. In conclusion, we present late onset of peritoneal recurrence of gastric cancer more than 20 years after primary surgery, and speculate on the mechanism of late-onset recurrence in our case.

## Background

Gastric cancer is a leading cause of tumor-related deaths, with approximately 50,000 individuals dying of gastric cancer each year in Japan. Death from gastric cancer is almost entirely caused by recurrent disease. Peritoneal recurrence represents one of the most common causes of failure after curative surgery for gastric cancer [1-4]. The median survival rate after peritoneal recurrence is only about 6 months [5]. The prognosis of peritoneal recurrence is so poor that standard treatment has not been established. Usually, peritoneal recurrence occurs within 5 years after surgery, especially during the first 2 years [5,6], and late-onset peritoneal recurrence of gastric cancer more than 10 years after surgery is extremely rare.

We report a case of peritoneal recurrence of gastric cancer that occurred more than 20 years after curative surgery, and review previous reports of these cases. Furthermore, by using immunohistochemical analysis, we also investigated how expression of several proteins, such as proliferating cell nuclear antigen (PCNA), Ki67 (cell proliferation), and p53 (resistance to chemoradiotherapy), differ between primary and secondary resection samples, and speculate on the mechanism of late-onset recurrence in our case.

## Case report

A 41-year-old man with no remarkable medical history was referred to our department with a two-month history of difficulty on swallowing and appetite loss. A barium study and endoscopic examination showed type 4 advanced gastric cancer in the lesser curvature of the upper body of the stomach. On admission, the tumor markers, such as carcinoembryonic antigen(CEA) and gastrointestinal cancer antigen 19-9 were within normal limits. He underwent total gastrectomy (D2 dissection) with splenectomy and distal pancreatectomy for advanced gastric cancer in the greater curvature of the corpus. Histological examination of the resected specimen revealed tubular adenocarcinoma of the moderately differentiated type with partial signet-ring cell carcinoma. The depth of tumor invasion was confirmed as exposed-serosal (se). There was mild lymphatic invasion (ly1), no venous invasion (v0), and lymph node metastasis (n2). The patient received adjuvant chemotherapy with oral 5-fluorouracil for 1 year, and was followed up for 5 years on an outpatient basis without any sign of recurrence.

**Figure 1:**
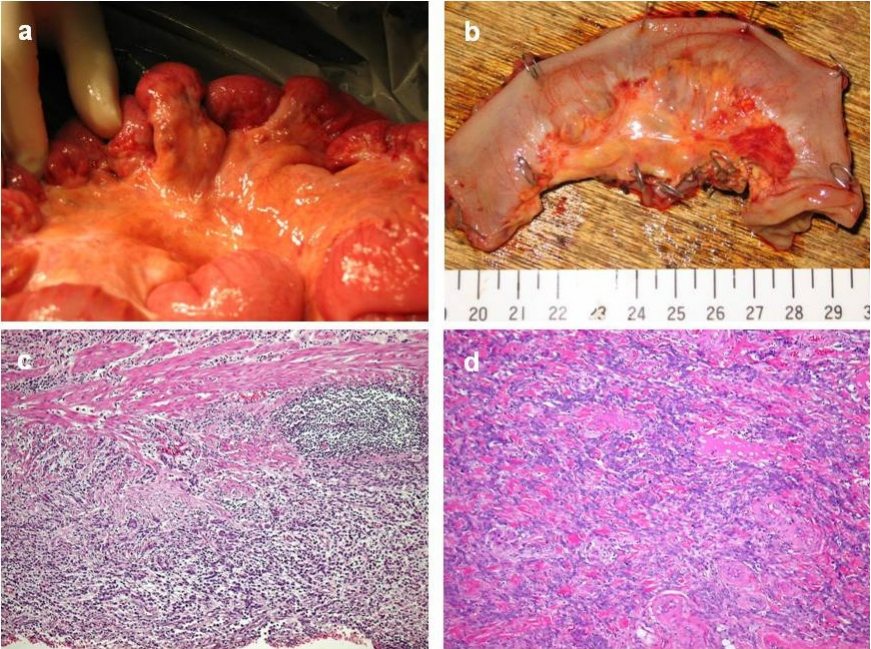
**At laparotomy, multiple white nodules (~10 mm) were observed within the mesentery proper (**a**)**. Resected specimens showed a whitish, hard nodule (12 × 10 mm) at the site of mesenteric attachment (**b**). Hematoxylin and eosin staining of primary cancer (**c**, × 100) and site of recurrence (**d**, × 100).

At the age of 61 years, 20 years after initial surgery, he was referred to our hospital with constipation, abdominal distention and lower abdominal pain. Abdominal computed tomography (CT) showed thickening of the intestinal wall, moderately accumulated ascites, and left hydronephrosis. Upper gastrointestinal fiberscope could demonstrate neither recurrent tumor nor another primary tumor. Radiographic contrast enema and colonoscopy could not evaluate the patient adequately because of extrinsic compression of the sigmoid colon. Tumor markers such as carcinoembryonic antigen, gastrointestinal cancer antigen 19-9, and alpha-fetoprotein were within normal limits. In contrast, cancer-related antigen 72-4 was elevated slightly. Laparotomy was performed because small-bowel obstruction could not be relieved by conservative therapy. At laparotomy, multiple white nodules (~10 mm) were observed within the mesentery proper (Figure 1a and 1b). Stenosis caused by these nodules was recognized in the descending and sigmoid colon. In addition, we checked other organs which contain remnant pancreas head, small intestine, colon and other intra-abdominal organs, and operative findings could not demonstrated primary other cancer. We diagnosed peritoneal dissemination from an unknown primary lesion, and diverting ileostomy with partial ileal resection was performed.

**Figure 2:**
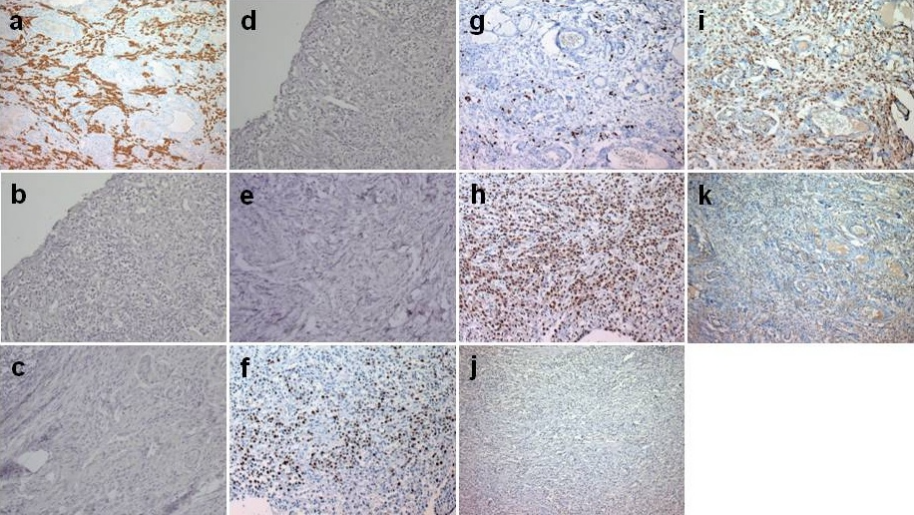
**Immunohistochemical staining of primary cancer and recurrent focus**. pan-CK at site of recurrence (**a**, × 100); CK-7 in primary (**b**, × 200) and recurrent (**c**, × 200) cancer; CK-20 in primary (**d**, × 200) and recurrent (**e**, × 200) cancer; Ki-67 in primary (**f**, × 200) and recurrent (**g**, × 200) cancer; PCNA in primary (**h**, × 200) and recurrent (**i**, × 200) cancer; and p53 in primary (**j**, × 100) and recurrent (**k**, × 100) cancer.

Histological examination for these white nodules revealed tubular adenocarcinoma with signet-ring cell carcinoma, which was similar to the pathological findings of the gastric tumor 20 years earlier (Figure 1c and 1d). Immunohistochemical staining using antibody to pancytokeratin (pan-CK; 1:50; NovoCastro, Newcastle, UK) showed that cancer cells were expressed in recurrent specimens (Figure 2a). The immunohistochemical staining for cytokeratin-7 (CK-7; 1:50; DAKO, Glostrup, Denmark) was not expressed both in primary and disseminated cancer cells (Figure 2b and 2c). In addition, immnohistochemical staining for CK-20 (1:25; DAKO, Glostrup, Denmark) was also not expressed both in primary and disseminated 6cancer cells (Figure 2d and 2e). The intensity of staining for Ki-67 (1:50; Zymed, San Francisco, CA, USA) and PCNA (1:100; DAKO, Glostrup, Denmark) was decreased in recurrent cancer cells in comparison to primary cancer specimens (Figure 2f-i). On the other hand, staining for p53 (1:100; DAKO) was more positive and diffuse in recurrent than primary cancer cells (Figure 2j and 2k).

Systemic reevaluation, including total colonoscopy and systemic computed tomography, could not confirm another primary tumor as the cause of the recurrence, therefore, peritoneal recurrence of gastric cancer more than 20 years after primary surgery was diagnosed. Follow-up systemic computed tomography at 1 year after the second operation could not showed primary other lesion responsible for peritoneal dissemination. The patient's general condition after secondary surgery took a gradual turn for the worse, despite 5-fluorouracil-based systemic chemotherapy, and he died at 62 years of age, 21 years and 7 months after initial gastrectomy.

## Discussion

Although sites of recurrence of gastric cancer include the peritoneum, liver, bone marrow and lymph nodes, peritoneal dissemination is the most common site of recurrence [7,8]. Peritoneal recurrence in gastric cancer has been reported in 17% of patients who underwent curative resection [5]. Especially in advanced gastric cancer, peritoneal recurrence is more frequent. Peritoneal recurrence of gastric cancer is thought to occur by spreading through the serosa or the lymphatic or vascular systems [4,9]. Then, peritoneal recurrence is involved closely with advanced invasion of the serosa and lymph node metastasis.

Shiraishi et al have compared early and late recurrence after gastrectomy for gastric cancer patients, and have reported that tumor size, lymphatic invasion, level of lymph node metastasis, stage of disease, and extent of lymph node dissection are the factors associated most significantly with early recurrence within 2 years after gastrectomy [10]. In contrast, Moon et al [11] have recently reported the changing pattern of prognostic indicators during 15 years' follow-up of advanced gastric cancer after gastrectomy and adjuvant chemotherapy. They have suggested that tumor factors, including stage, were clinical prognostic indicators within 5 years post-gastrectomy, but there were no such indicators after 10 years.

Previously, there have been only three reported cases in which peritoneal recurrence occurred more than 10 years after primary resection (Table. 1). Aihara et al [12] have demonstrated peritoneal recurrence of primary advanced gastric cancer, with serosal invasion (se) accompanied with lymph node metastasis (n2), 12 years after curative resection. Moon et al [11] have reported two cases of peritoneal recurrence with or without distant metastasis at 12-14 years after radical gastrectomy. In our case, late-onset peritoneal recurrence was detected more than 20 years after curative gastrectomy, regardless of the primary advanced stage with serosal invasion and lymph node metastasis. All reviewed cases, including the present one, support the possibility that late-onset peritoneal recurrence of gastric cancer after more than 10 years has no prognostic indicators. This is because these cases of peritoneal recurrence were from advanced gastric cancer, and the mechanism of such late-onset recurrence of gastric cancer remains unclear.

**Table 1:** Late onset peritoneal recurrence of gastric cancer over 10 years after curative resection

No	Author	Age	Sex	TNM stage at initial operation	Recurrence free survival	With or without distant metastasis
1	Moon YW, 2007^11)^	Unknown	Unknown	II or IIIA	12-14 years	With distant metastasis

2	Moon YW, 2007^11)^	Unknown	Unknown	II or IIIA	12-14 years	Without distant metastasis

3	Aihara R, 2007^12)^	58-years-old	Male	IIIB	12 years	Without distant metastasis

4	Our Case, 2010	61-years-old	Male	IIIB	20 years	Without distant metastasis

We also assessed both the organ specificity of disseminated lesion and the mechanism of late-onset recurrence of gastric cancer by immunohistochemistry using pan-CK, CK-7, and CK-20 as a indicator of epithelial differentiation [13-16], p53 as a chemo-radioresistance marker [17], PCNA, and Ki-67 as a cell proliferation marker [18,19]. CK-7 and CK-20 have been used for immunophenotyping of metastases and primary adenocarcinomas a the study by Wauters et al [14]. In our case, primary gastric cancer showed the same CK-7/CK-20 expression pattern as disseminated lesion. The CK-7^-^/CK-20^- ^pattern was reported to be found from 10% to 25% of gastric carcinoma [15,16], and these data might support the diagnosis of the peritoneal dissemination of gastric cancer 20 years after curative resection. Furthermore, recurrent cancer cells expressed Ki-67 and PCNA at a lower level than the primary lesion in each specimen. On the other hand, staining with antibodies against p53 revealed that recurrent cancer cells had higher expression of mutant p53 than primary cancer cells did. The clinical course corroborated these findings, which suggest that recurrent cancer cells have slow proliferation and are resistant to chemotherapy.

## Conclusions

In conclusion, we report an extremely rare case of peritoneal recurrence detected 20 years after curative surgery for advanced gastric cancer. Regardless of the advanced stage, peritoneal recurrence should be considered as one of the recurrence patterns of gastric cancer during long follow-up in patients for more than 10 years after curative gastrectomy.

## Consent

Written informed consent was obtained from the patient for publication of this case report and any accompanying images. A copy of the written consent is available for review by the Editor-in-Chief of this journal.

## Competing interests

The authors declare that they have no competing interests.

## Authors' contributions

YO did the literature search and writing of the manuscript. YT and YI revised the manuscript critically for important intellectual content. SS, MK, KT, MY, and CM carried out histopathological analyses and review of the manuscript. MK is guarantor of the paper. All authors read andapproved the final manuscript.
